# Design, Validation and Comparison of Path Following Controllers for Autonomous Vehicles

**DOI:** 10.3390/s20216052

**Published:** 2020-10-24

**Authors:** Xing Yang, Lu Xiong, Bo Leng, Dequan Zeng, Guirong Zhuo

**Affiliations:** 1 School of Automotive Studies, Tongji University, Shanghai 201804, China; yang_xing@tongji.edu.cn (X.Y.); xiong_lu@tongji.edu.cn (L.X.); zdq1610849@126.com (D.Z.); zhuoguirong@tongji.edu.cn (G.Z.); 2 Clean Energy Automotive Engineering Center, Tongji University, Shanghai 201804, China; 3 Postdoctoral Station of Mechanical Engineering, Tongji University, Shanghai 201804, China

**Keywords:** autonomous vehicles, path following, speed tracking, model predictive control, nonlinear active disturbance rejection control

## Abstract

As one of the core issues of autonomous vehicles, vehicle motion control directly affects vehicle safety and user experience. Therefore, it is expected to design a simple, reliable, and robust path following the controller that can handle complex situations. To deal with the longitudinal motion control problem, a speed tracking controller based on sliding mode control with nonlinear conditional integrator is proposed, and its stability is proved by the Lyapunov theory. Then, a linear parameter varying model predictive control (LPV-MPC) based lateral controller is formulated that the optimization problem is solved by CVXGEN. The nonlinear active disturbance rejection control (ADRC) method is applied to the second lateral controller that is easy to be implemented and robust to parametric uncertainties and disturbances, and the pure pursuit algorithm serves as a benchmark. Simulation results in different scenarios demonstrate the effectiveness of the proposed control schemes, and a comparison is made to highlight the advantages and drawbacks. It can be concluded that the LPV-MPC has some trouble to handle uncertainties while the nonlinear ADRC performs slight worse tracking but has strong robustness. With the parallel development of the control theory and computing power, robust MPC may be the future direction.

## 1. Introduction

Autonomous driving technology has become a research and development hotspot recently, since it has great potential to improve active safety, alleviate traffic congestion, and reduce energy consumption [1,2]. It was reported that the driver’s mistakes contribute entirely or partially to nearly 90% of road accidents [3]. The autonomous system is more reliable and faster to react than human drivers, so it can handle critical scenarios that human drivers find challenging or lack the ability to navigate, such as emergency obstacle avoidance [4]. On the other hand, under the perspective of networked control, the platooning of autonomous vehicles provides an effective way to increase traffic capacity and fuel efficiency [5]. Therefore, numerous contests of autonomous vehicles have been carried out to promote technological development, such the DARPA (Defense Advanced Research Projects Agency) Challenges in the USA [6] and the Intelligent Vehicle Future Challenges in China [7], etc. Simultaneously, the research of autonomous vehicles is booming and highly competitive in the industry. The Waymo and Cruise autonomous driving cars are typical commercial products that have attracted wide attention [8].

The key technologies of autonomous vehicles consist of environment perception, decision making, motion planning, and vehicle motion control, and the objective of vehicle motion control can be divided into path stabilization and trajectory stabilization [1]. The difference between them is that the reference trajectory is independent of time in the path following problem, while the trajectory tracking is related to time. In this paper, we focus on the path following issue of autonomous vehicles, which manipulates steering wheel to guide the vehicle to track a desired path. The path can be generated offline based on a priori or online through a high-level planning layer. The objective of path following is to realize accurate and smooth tracking while the vehicle stability is guaranteed. Besides, robustness is also a key issue in control design considering the high nonlinearity of the vehicle dynamics and the parametric uncertainties and disturbances. Many algorithms have been applied to deal with path following, including the pure pursuit method (PPM) [9], sliding mode control (SMC) [10,11], and model predictive control (MPC) [12,13,14], etc. However, there are still some challenges for practical real-time implementation although the previous research achievements were successful to some extent. For instance, MPC controllers may suffer from heavy computational burden due to online optimization, especially for the nonlinear MPC. Moreover, it is difficult to accurately model the vehicle dynamics at the handling limits. On the other hand, make everything as simple as possible is necessary for implementation in practice. Therefore, it is expected to design a simple, reliable, and robust path tracking controller.

In this paper, we design, validate, and compare three path following schemes to track a predetermined path, using linear parameter varying model predictive control, nonlinear active disturbance rejection control [15], and the pure pursuit method, respectively. The main contributions of this paper are summarized as follows:

- A longitudinal speed controller based on sliding mode control with nonlinear conditional integrator is proposed to realize the longitudinal motion control of autonomous vehicle during the path following process. It is well known that sliding mode controllers suffer from chattering. To solve this problem, we adopt the saturation function to replace the sign function, meanwhile, a nonlinear integral action is introduced to achieve zero steady-state error and improve the transient performance, which can also avoid integral divergence. Stability analysis is given to prove that the equilibrium point is globally asymptotically stable.
- The lateral controllers based on LPV-MPC (Linear Parameter Varying Model Predictive Control), ADRC (Active Disturbance Rejection Control), and PPM (Pure Pursuit Method)are designed, respectively. First, because the MPC can exploit available preview information and handles constraints, an MPC lateral control-based strategy that considers the soft constraints of the sideslip angle of the steer wheel is formulated based on the error dynamics model, which adopts the CVXGEN [16] solver to improve computational efficiency. Second, the nonlinear ADRC is applied to develop the lateral controller due to the simple control structure and good robustness, which is also largely model independent. Finally, the pure pursuit method using the geometric model is provided as a benchmark.
- Multiple simulations in different scenarios are conducted to validate the effectiveness and capability of the proposed longitudinal speed controller and lateral path following controllers. A comparison of the three mentioned lateral controllers is made to highlight the advantages and drawbacks of each approach in path following. Finally, the possible development direction in the future is given.

The rest of this paper is organized as follows: Section 2 introduces the state of the art. Section 3 presents the system model and the problem definition. Section 4 explains the design procedure for the longitudinal speed controller. Section 5 presents the lateral controllers design of LPV-MPC, ADRC, and PPM, respectively. Section 6 shows the results and analysis. Finally, the paper is concluded in Section 7.

## 2. Related Work

Extensive work has been done to vehicle motion control of autonomous vehicles. In this section, we provide a brief review of the state of the art. Vehicle motion control can be divided into longitudinal control and lateral control. Further, lateral control methods can be divided into geometry based, kinematics based, and dynamics based.

The longitudinal control manipulates drive and brake actuators to guide the autonomous vehicle to track a desired speed profile. The main control methods can be roughly classified into three categories: (1) Model-free control. The system dynamics are regarded as a black-box, and the control commands are generated from tracking errors only, e.g., the proportional-integral-derivative (PID) design [17]. (2) Model-based feedback control. A discrete-time preview speed controller was developed considering future desired speed and road slope information, which holds the advantage of prediction and to reduce to computational load as well compared to MPC [18]. Gerdes et al. utilized a multiple-surface sliding mode control to realize speed and space control [19]. To improve tracking performance under model uncertainties and external disturbances, a time-varying parameter adaptive speed control algorithm is proposed [20]. (3) Model-based optimization. The model predictive control framework was applied in a 16-bit micro controller, which can exploit available preview information [21]. However, it may suffer from high computational load, especially for the nonlinear MPC. Improving the computational efficiency is a key issue for MPC application.

The lateral control manipulates the steering actuator to deal with the path following problem generally, and the direct yaw moment control can be used as an additional approach for distributed drive vehicles and vehicles equipped with differential braking. According to the difference of the system model, path tracking control methods can be roughly classified into three categories: (1) Geometry based. It used the geometric relationship between the vehicle and reference. Classical algorithms include the pure pursuit method and Stanley algorithm [17], which were used in the DARPA Challenge vehicle. Such methods are simple and work well in many situations, but may have trouble on tight corners and at high speed due to a lack of consideration for vehicle dynamics. (2) Kinematics based. It is usually assumed that the autonomous vehicle satisfies non-holonomic constraint, in other words, the side slip angle is assumed to be zero. Aiming at the path tracking problem of the non-holonomic mobile robot, the kinematics model is transformed into the chain type by state and input feedback transformation, and a smooth time-varying feedback control law was given [9]. (3) Dynamics based. In order to obtain higher precision under the conditions of high speed and large curvature, it is necessary to consider the vehicle dynamics in the control design. A nested PID steering control scheme was proposed that the outer loop is used to compute yaw rate reference based on lateral offset and then the inner loop tracks the desired yaw rate [22]. Xu et al. presented a preview steering control algorithm, which formulated the path tracking issue as an augmented optimal control problem with dynamic disturbance [23]. Tagne et al. analyzed and compared three lateral nonlinear adaptive controllers, including the higher order sliding mode controller, the immersion and invariance controller, and the passivity-based controller [24]. MPC has become a popular technique that enables the vehicle to act ahead of time and handle constraints more conveniently. Falcone et al. demonstrated the effectiveness of linear MPC-based steering controller with a speed up to 21 m/s on a slippery road [25], and proposed a control scheme by a combined use of breaking and steering [26]. The integration of path tracking, vehicle stabilization, and collision avoidance had been investigated by considering stability envelope and environmental envelope in state and output constraints of MPC [27,28]. To safely navigate highly dynamic scenarios, the nonlinear model predictive controller also had been designed and validated [29]. It should be noted that appropriate tradeoffs between model fidelity and computation should be solved with caution [30]. Hu et al. [31] proposed a robust $H_{\infty }$ output-feedback control strategy considering the parametric uncertainties and external disturbances. For the similar purpose, a linear active disturbance rejection control scheme is proposed for lane keeping problem [32]. Compared to this work, the nonlinear active disturbance rejection is used in this paper to improve the transient performance.

## 3. Systems Description

**Figure 1:**
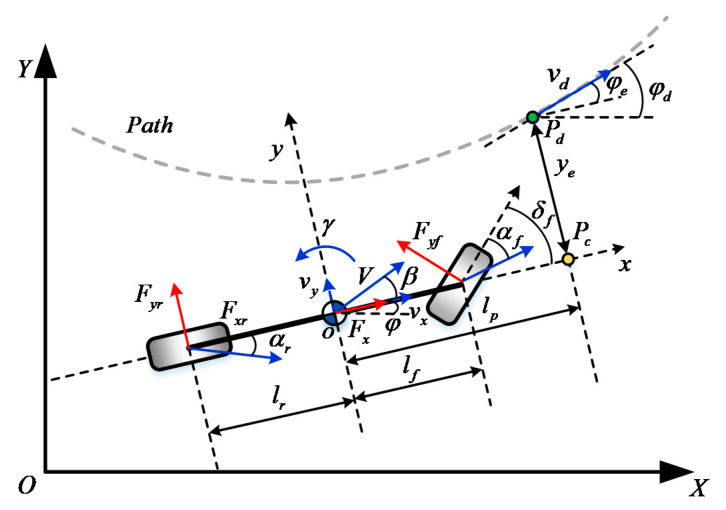
Schematic of path following system. OXY is the global coordinate system and oxy is the vehicle body-fixed local coordinate system.

In order to describe the motion of an autonomous vehicle, the system dynamics of path following is defined, as illustrated in Figure 1. A preview lateral model is utilized here, where $p_{c}$ is the preview control point and $p_{d}$ is the reference path point.

### 3.1. Longitudinal Vehicle Dynamics

Considering a vehicle moving on an inclined road, a force balance along the vehicle longitudinal axis yields:

(1) $$m({\dot{v}}_{x}-v_{y}\gamma )=F_{x}-F_{yf}\sin\delta_{f}-F_{r}-F_{w}-F_{g},$$

where m is the vehicle mass, $v_{x}$ and $v_{y}$ are the vehicle longitudinal and lateral velocities, γ is the vehicle yaw rate, $F_{x}$ is the total tire force along the vehicle longitudinal axis, $F_{yf}$ is the lateral tire force of the front steering wheel, $\delta_{f}$ is the front wheel steering angle, $F_{r}$ is the rolling resistance force, $F_{w}$ is the aerodynamic force, and $F_{g}$ is the gravitational force component generated by road grade.

The wheel dynamics can be expressed as the following equation:

(2) $$I_{\omega }{\dot{\omega }}_{ij}=T_{ij}-F_{xij}R,\;ij=fl,fr,rl,rr,$$

where $I_{\omega }$ is the wheel inertia, $\omega_{ij}$ is the wheel angular velocity and the subscript represents the four wheel of the vehicle, $F_{xij}$ is the longitudinal tire force, R is the wheel rolling radius.

### 3.2. Lateral Vehicle Dynamics

The objective of lateral control design is to eliminate the path error while guaranteeing vehicle stability. As shown in Figure 1, it is desirable to both eliminate the lateral error $y_{e}$ and heading angle error $\varphi_{e}$ at the preview point by manipulating the control input steering angle $\delta_{f}$.

The preview lateral kinematics can be modeled as:

(3) $$\left\{ \begin{array}{l} {\dot{y}}_{e}=v_{x}\varphi_{e}-v_{x}\beta -l_{p}\gamma \\ {\dot{\varphi }}_{e}=v_{x}\kappa -\gamma \end{array}\right.,$$

where β is the sideslip angle, $l_{p}$ is the preview distance, κ is the path curvature.

The 2DOF vehicle dynamics model is used to design the lateral controller, which can be expressed as:

(4) $$\left\{ \begin{array}{l} \dot{\beta }=\frac{-2(C_{\alpha f}+C_{\alpha }{}_{r})}{mv_{x}}\beta +\left(\frac{-2(C_{\alpha f}l_{f}-C_{\alpha }{}_{r}l_{r})}{mv_{x}^{2}}-1\right)\gamma +\frac{2C_{\alpha f}}{mv_{x}}\delta_{f}\\ \dot{\gamma }=\frac{-2(C_{\alpha f}l_{f}-C_{\alpha }{}_{r}l_{r})}{I_{z}}\beta +\frac{-2(C_{\alpha f}l_{f}^{2}+C_{\alpha }{}_{r}l_{r}^{2})}{I_{z}v_{x}}\gamma +\frac{2C_{\alpha f}l_{f}}{I_{z}}\delta_{f} \end{array}\right.,$$

where $C_{\alpha f}$ and $C_{\alpha }{}_{r}$ denote the cornering stiffness of the single front tire and rear tire, $I_{z}$ is the yaw moment of inertia, $l_{f}$ and $l_{r}$ are the distance from the front axle and rear axle to the center of gravity, respectively.

The combined dynamics (3) and (4) can be written in the state space form, with the states $x={\left[\begin{array}{cccc} y_{e} & \varphi_{e} & \beta & \gamma \end{array}\right]}^{T}\in {\mathbb{R}}^{4}$, the control input $u=\delta_{f}\in \mathbb{R}$, the system outputs $y={\left[\begin{array}{cc} y_{e} & \varphi_{e} \end{array}\right]}^{T}\in {\mathbb{R}}^{2}$, and the measurable external disturbance w=κ(t)∈ℝ:

(5) $$\begin{array}{l} \dot{x}=Ax+Bu+Dw\\ y=Cx \end{array},$$

in which:

$$\begin{array}{l} A=\left[\begin{array}{cccc} 0 & v_{x} & -v_{x} & -l_{p}\\ 0 & 0 & 0 & -1\\ 0 & 0 & \frac{-2(C_{\alpha f}+C_{\alpha }{}_{r})}{mv_{x}} & \frac{-2(C_{\alpha f}l_{f}-C_{\alpha }{}_{r}l_{r})}{mv_{x}^{2}}-1\\ 0 & 0 & \frac{-2(C_{\alpha f}l_{f}-C_{\alpha }{}_{r}l_{r})}{I_{z}} & \frac{-2(C_{\alpha f}l_{f}^{2}+C_{\alpha }{}_{r}l_{r}^{2})}{I_{z}v_{x}} \end{array}\right],B=\left[\begin{array}{c} \begin{array}{cc} 0 & 0 \end{array}\\ \begin{array}{c} \begin{array}{c} \begin{array}{cc} 0 & 0 \end{array}\\ \begin{array}{cc} \frac{2C_{\alpha f}}{mv_{x}} & 0 \end{array} \end{array}\\ \begin{array}{cc} \frac{2C_{\alpha f}l_{f}}{I_{z}} & \frac{1}{I_{z}} \end{array} \end{array} \end{array}\right]\\ C=\left[\begin{array}{cccc} \begin{array}{c} 1\\ 0 \end{array} & \begin{array}{c} 0\\ 1 \end{array} & \begin{array}{c} 0\\ 0 \end{array} & \begin{array}{c} 0\\ 0 \end{array} \end{array}\right],D=\left[\begin{array}{c} 0\\ v_{x}\\ 0\\ 0 \end{array}\right] \end{array}.$$

## 4. Longitudinal Speed Controller Design

**Figure 2:**
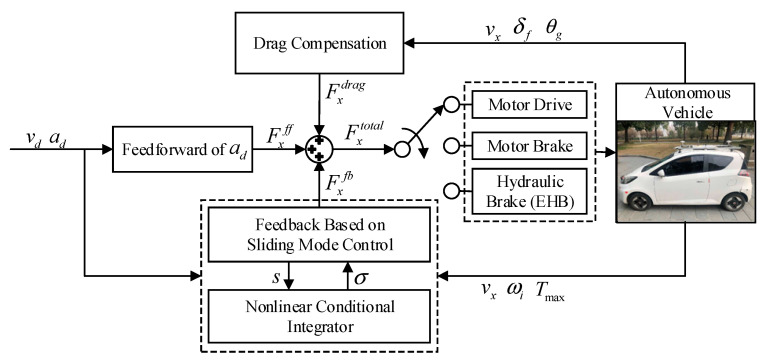
Block diagram of longitudinal speed controller via the sliding mode control with nonlinear conditional integrator.

The objective of longitudinal speed control is to track the desired speed profile accurately and smoothly, by manipulating the motor and electro-hydraulic brake system (EHB) in this paper. To improve the robustness to parametric uncertainties and provide disturbance rejection, a speed tracking controller via sliding mode control with nonlinear conditional integrator is developed. The control framework is shown in Figure 2.

**Figure 3:**
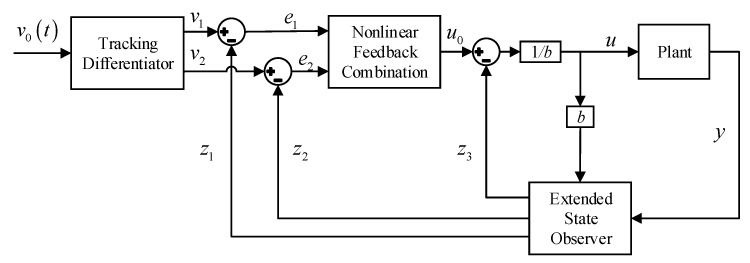
Control block diagram of the nonlinear ADRC.

From the control block diagram in Figure 3, it can be seen that the longitudinal speed controller consists of feedforward and feedback components. The feedforward term uses the desired acceleration profile to calculate the acceleration resistance, and compensates the road resistance according to vehicle operating conditions simultaneously. In the feedback control term, the sliding mode control method is adopted. Note that integral action is introduced in the sliding mode controller to ensure asymptotic tracking.

### 4.1. Feedforward of Speed Controller

#### 4.1.1. Feedforward from Reference Acceleration

According to the desired longitudinal acceleration $a_{d}$ at the reference point generated offline or provided by high-level planner, feedforward longitudinal force from reference acceleration $F_{x}^{ff}$ can be calculated from Newton’s second law as follows:

(6) $$F_{x}^{ff}=ma_{d}.$$

#### 4.1.2. Drag Compensation

As shown in Equation (1), the road resistance concludes the resistance force from the vehicle’s rolling $F_{r}$, aerodynamic force $F_{w}$, road grade $F_{g}$, and the longitudinal component from the front axle turning $F_{t}$. The expression of each resistance is given as follows:

(7) $$\begin{array}{l} F_{r}=mgf\\ F_{w}=\frac{C_{D}Av_{x}^{2}}{21.15}\\ F_{g}=mg\sin\theta_{g}\\ F_{t}=F_{yf}\sin\delta_{f}\approx \frac{ml_{f}\left|a_{y}\tan\delta_{f}\right|}{l} \end{array},$$

where f is the rolling resistance coefficient, $C_{D}$ is the air resistance coefficient, A is the frontal area of the vehicle, $\theta_{g}$ is the road grade measured or estimated, $a_{y}$ is the lateral acceleration. The calculation of $F_{t}$ uses a steady state assumption and assumes that the front lateral force is proportional to the front static vertical load.

Then, the drag compensation is obtained from:

(8) $$F_{x}^{drag}=F_{r}+F_{w}+F_{g}+F_{t},$$

The total feedforward longitudinal torque is expressed as:

(9) $$T_{ff}=\left(F_{x}^{ff}+F_{x}^{drag}\right)R.$$

### 4.2. Feedback of Speed Controller

Robust asymptotic tracking can be achieved by the sliding mode control, therefore, it is applied here. To simplify the longitudinal dynamic model, assuming that the lateral velocity is zero, then we can get:

(10) $$m{\dot{v}}_{x}=F_{x}-F_{r}-F_{w}-F_{g}-F_{t}.$$

Using the pure rolling assumption that $\omega_{ij}=v_{xij}/R$, the wheel dynamics in Equation (2) can be rewritten as:

(11) $$I_{\omega i}\frac{{\dot{v}}_{xij}}{R}=T_{ij}-F_{xij}R\;ij=fl,fr,rl,rr.$$

Combining Equations (10) and (11):

(12) $$\left(m+\frac{\sum I_{\omega ij}}{R^{2}}\right){\dot{v}}_{x}=\frac{T}{R}-F_{r}-F_{w}-F_{g}-F_{t},\;ij=fl,fr,rl,rr,$$

where T is total control input torque, and $T=T_{ff}+T_{fb}$, $T_{fb}$ is the feedback control input torque.

Define the tracking error $v_{e}=v_{x}-v_{d}$, $v_{d}$ is the desired speed. Then, the tracking problem can be turned into a stabilization problem. Considering the resistance estimation error term, the stabilization system can be expressed as follows by combining Equations (6)–(10):

(13) $$\left(m+\frac{\sum I_{\omega ij}}{R^{2}}\right){\dot{v}}_{e}=T_{fb}+\Delta \left(\cdot \right),\;ij=fl,fr,rl,rr,$$

where Δ(⋅) is the bounded resistance estimation error, and it is considered that slew rate is small.

It is typical to choose the sliding surface as $s=v_{e}$ for a first order system, the sliding mode control law can be expressed as:

(14) $$T_{fb}=-\left(m+\frac{\sum I_{\omega ij}}{R^{2}}\right)k\mathrm{sgn}(s)=-k_{p}\mathrm{sgn}(v_{e}),\;ij=fl,fr,rl,rr,$$

with sufficiently large control gain $k_{p}$, ensures that $s\dot{s}\leq -p_{0}\left|s\right|<0$, for all s≠0. While ideal sliding mode control achieves zero steady-state error, it is well known that, in practices, sliding mode controllers suffer from chattering due to switch delays and un-modeled dynamics. To eliminate chattering, we use the saturation function sat(⋅) to replace the sign function sgn(⋅), but it can guarantee only ultimate boundness with respect to a compact set, which can be made arbitrarily small by decreasing the thickness ε of boundary layer. However, a too small value of ε will again induce chattering due to non-ideal effects:

(15) $$T_{fb}=-k_{p}sat(\frac{s}{\varepsilon }),$$

where sat(⋅) is defined as:

(16) $$sat(y)=\left\{ \begin{array}{l} y,\;\;\;\left|y\right|\leq 1\\ \mathrm{sgn}(y),\;\left|y\right|>1\; \end{array}\right..$$

Zero steady-state error can be achieved by including integral action in the controller. This was done by Khalil [33] by augmenting the system with an integrator driven by the tracking error: $\dot{\sigma }=v_{e}$. The sliding surface is taken as:

(17) $$s=k_{0}\sigma +v_{e},$$

where the positive constant $k_{0}$ is chosen such that the system dynamics on sliding surface is controlled by ${\dot{v}}_{e}=-k_{0}v_{e}$.

In this paper, the linear integrator is replaced by a nonlinear conditional integrator to avoid integral divergence and improve the transient performance:

(18) $$\dot{\sigma }=-k_{0}\sigma +\varepsilon sat(\frac{s}{\varepsilon }).$$

At the actuator level, the electric brake is used for slight braking case to improve response speed and control accuracy, and the hydraulic brake compensation is used for heavy braking case.

### 4.3. Stability Analysis

To analyze the stability performance of the sliding mode controller with the conditional integrator, it is carried out from two aspects of inside the boundary layer and outside the boundary layer.

#### 4.3.1. Inside the Boundary Layer ($\left|s\right|=\left|k_{0}\sigma +v_{e}\right|<\varepsilon$)

Substituting Equation (15) into Equation (13), we can have:

(19) $$\left\{ \begin{array}{l} {\dot{v}}_{e}=\frac{1}{m+\frac{\sum I_{\omega ij}}{R^{2}}}\left(-\frac{k_{p}}{\varepsilon }v_{e}-\frac{k_{p}k_{0}}{\varepsilon }\sigma +\Delta \left(\cdot \right)\right),\;ij=fl,fr,rl,rr\\ \dot{\sigma }=v_{e} \end{array}\right..$$

The closed-loop system has a unique equilibrium point $\left({\bar{v}}_{e},\bar{\sigma }\right)$ at $\left(0,\frac{\varepsilon }{k_{p}k_{0}}\Delta \left(v_{d}\right)\right)$.

Selecting a Lyapunov candidate function:

(20) $$V=\frac{1}{2}{\tilde{s}}^{2}+\frac{1}{2}{\tilde{\sigma }}^{2},$$

where $\tilde{\sigma }=\sigma -\bar{\sigma },\tilde{s}=s-\bar{s}$ and $\bar{s}={\bar{v}}_{xe}+k_{i}\bar{\sigma }=k_{i}\bar{\sigma }$.

Differentiating the left and right sides of Equation (20), it can be expressed as:

(21) $$\begin{array}{lll} \dot{V} & = & \tilde{s}\dot{\tilde{s}}+\tilde{\sigma }\dot{\tilde{\sigma }}=\tilde{s}\dot{s}+\tilde{\sigma }\dot{\sigma }=\tilde{s}({\dot{v}}_{e}+k_{0}\dot{\sigma })+\tilde{\sigma }v_{e}\\ & = & \frac{\tilde{s}}{m+\frac{\sum I_{\omega ij}}{R^{2}}}\left(-k_{p}\frac{v_{e}+k_{0}\sigma }{\varepsilon }+\Delta \left(v_{x}\right)\right)+\tilde{s}k_{0}v_{e}+\tilde{\sigma }v_{e},\;ij=fl,fr,rl,rr\\ & = & \frac{\tilde{s}}{m+\frac{\sum I_{\omega ij}}{R^{2}}}\left(-k_{p}\frac{\tilde{s}+\bar{s}}{\varepsilon }+\Delta \left(v_{x}\right)\right)+\tilde{s}k_{0}v_{e}+\tilde{\sigma }v_{e},\;ij=fl,fr,rl,rr \end{array}.$$

Substituting $v_{e}=s-k_{0}\sigma =\tilde{s}-k_{0}\tilde{\sigma }$ into Equation (16), then the derivative of the Lyapunov function candidate can be rewritten as follows:

(22) $$\dot{V}=-\left(\frac{k}{\varepsilon }-k_{0}\right){\tilde{s}}^{2}+\frac{\tilde{s}}{m+\frac{\sum I_{\omega ij}}{R^{2}}}\left(-k_{p}\frac{\bar{s}}{\varepsilon }+\Delta \left(v_{x}\right)\right)-\left(k_{0}{}^{2}-1\right)\tilde{s}\tilde{\sigma }-k_{0}{\tilde{\sigma }}^{2}.$$

Define a function $f\left(x\right)=\frac{\Delta \left(x\right)}{m+\frac{\sum I_{\omega ij}}{R^{2}}},\;ij=fl,fr,rl,rr$, when the continuous Lipschitz continuity condition is satisfied, it can be obtained that:

(23) $$\left|f\left(v_{x}\right)-f\left(v_{d}\right)\right|\leq L\left|v_{e}\right|=L\left|\tilde{s}-k_{0}\tilde{\sigma }\right|\leq L\left|\tilde{s}\right|+Lk_{0}\left|\tilde{\sigma }\right|,$$

where L is the Lipschitz constant.

Combining Equation (23) and $\bar{s}=k_{0}\bar{\sigma }=\frac{\varepsilon }{k_{p}}\Delta \left(v_{d}\right)$, Equation (21) can be expressed as:

(24) $$\begin{array}{ll} \dot{V} & =-\left(\frac{k}{\varepsilon }-k_{0}\right){\tilde{s}}^{2}+\tilde{s}\left(\frac{\Delta \left(v_{x}\right)-\Delta \left(v_{d}\right)}{m+\frac{\sum I_{\omega ij}}{R^{2}}}\right)-\left(k_{0}{}^{2}-1\right)\tilde{s}\tilde{\sigma }-k_{0}{\tilde{\sigma }}^{2}\\ & \leq -\left(\frac{k}{\varepsilon }-k_{0}\right){\tilde{s}}^{2}+\left|\tilde{s}\right|\left(L\left|\tilde{s}\right|+Lk_{0}\left|\tilde{\sigma }\right|\right)-\left(k_{0}{}^{2}-1\right)\tilde{s}\tilde{\sigma }-k_{0}{\tilde{\sigma }}^{2}\\ & =-\left(\frac{k}{\varepsilon }-k_{0}-L\right){\tilde{s}}^{2}+\left(Lk_{0}+1-k_{0}{}^{2}\right)\left|\tilde{s}\right|\left|\tilde{\sigma }\right|-k_{0}{\tilde{\sigma }}^{2}\\ & =-\left(\begin{array}{cc} \left|\tilde{\sigma }\right| & \left|\tilde{s}\right| \end{array}\right)\left(\begin{array}{cc} k_{0} & -\frac{Lk_{0}+1-k_{0}{}^{2}}{2}\\ -\frac{Lk_{0}+1-k_{0}{}^{2}}{2} & \frac{k}{\varepsilon }-k_{0}-L \end{array}\right)\left(\begin{array}{c} \left|\tilde{\sigma }\right|\\ \left|\tilde{s}\right| \end{array}\right) \end{array}.$$

If the inequality $k_{0}>0$ and $k_{0}\left(\frac{k}{\varepsilon }-k_{0}-L\right)-{\left(\frac{Lk_{0}+1-k_{0}{}^{2}}{2}\right)}^{2}>0$ are both satisfied, $\dot{V}$ is negative definite. Therefore, the equilibrium point is globally asymptotically stable if the control parameters satisfy the following equation:

(25) $$\left\{ \begin{array}{l} k_{0}>0\\ 0<\varepsilon <{\left[\frac{k_{0}}{k}+\frac{L}{k}+\frac{1}{kk_{0}}{\left(\frac{Lk_{0}+1-k_{0}{}^{2}}{2}\right)}^{2}\right]}^{-1} \end{array}\right..$$

#### 4.3.2. Outside the Boundary Layer ($\left|s\right|=\left|k_{0}\sigma +v_{e}\right|\geq \varepsilon$)

Substituting Equation (15) into Equation (13), the control law outside the boundary layer can be expressed as:

(26) $$\left\{ \begin{array}{l} T_{fb}=-k_{p}\mathrm{sgn}\left(s\right)\\ \dot{\sigma }=-k_{0}\sigma +\varepsilon \mathrm{sgn}\left(s\right) \end{array}\right..$$

It can be seen that the feedback control input converges to $-k_{p}\mathrm{sgn}\left(s\right)$, the value of $\dot{\sigma }$ converges to zero, and σ becomes εsgn(s). Thus, the controller can prevent integral action from divergence. Furthermore, when ${\dot{v}}_{d}\mathrm{sgn}\left(s\right)>0$ is satisfied, it reaches the set {|s|≤ε} in finite time and remains inside thereafter.

## 5. Lateral Path Following Controllers Design

To obtain a simple, reliable, and robust path following controller that can handle complex situations, three lateral control schemes are proposed and compared. First, a LPV-MPC lateral control-based strategy is designed due to its ability of preview and handles constraints. Second, considering the simple control structure and good robustness, the nonlinear ADRC is applied to develop the second lateral controller. The third controller is based on the pure pursuit method, which is provided as a benchmark.

### 5.1. LPV-MPC Controller

Despite nonlinear MPC is attractive because of its ability to capture the nonlinearity of vehicle dynamics, the model complexity limited real-time implementation. Here, a LPV-MPC control law is designed.

#### 5.1.1. Vehicle Model Discretization and Augmentation

The continuous vehicle model Equation (5) is discretized at each time step with a zero-order hold approximation that assumes constant continuous inputs over the duration of each discrete time step. The resulting discretized vehicle model is:

(27) $$\begin{array}{l} x\left(k+1\right)=A_{k}x\left(k\right)+B_{k}u\left(k\right)+D_{k}w\left(k\right)\\ y\left(k\right)=C_{k}x\left(k\right) \end{array},$$

where $A_{k}=I_{n}+A_{t}T_{s},B_{k}=B_{t}T_{s},D_{k}=D_{t}T_{s},C_{k}=C_{t}$, $T_{s}$ is the sampling time.

To eliminate the steady-state error, we change the control input u(k) to the rate of change of the input Δu(k)=u(k)−u(k−1) that can achieve the steady-state performance like an integral action, and augment the state vector as $\xi \left(k\right)={\left[\begin{array}{ccc} x\left(k\right) & u\left(k-1\right) & w\left(k-1\right) \end{array}\right]}^{T}$. Thus, the augmented system model becomes:

(28) $$\begin{array}{l} \xi \left(k+1\right)=A\xi \left(k\right)+B\Delta u\left(k\right)\\ y\left(k\right)=C\xi \left(k\right) \end{array},$$

in which:

$$A=\left[\begin{array}{ccc} A_{k} & B_{k} & D_{k}\\ 0 & I & 0\\ 0 & 0 & 1 \end{array}\right],B=\left[\begin{array}{c} B_{k}\\ I\\ 0 \end{array}\right],C=\left[\begin{array}{ccc} C_{k} & 0 & 0 \end{array}\right].$$

#### 5.1.2. MPC Problem Formulation

Assuming that the current moment is k, the signals ξ(k+j|k), Δu(k+j|k), and y(k+j|k) represent the future value of the states, inputs, and outputs respectively. Considering the prediction based on the discrete state space model (28), the predict states can be obtained by iteration:

(29) $$\left\{ \begin{array}{lll} \xi \left(k+1|k\right) & = & A\xi \left(k\right)+B\Delta u\left(k|k\right)\\ \xi \left(k+2|k\right) & = & A\xi \left(k+1|k\right)+B\Delta u\left(k+1|k\right)\\ & = & A^{2}\xi \left(k\right)+AB\Delta u\left(k|k\right)+B\Delta u\left(k+1|k\right)\\ & \vdots & \\ \xi \left(k+N_{p}|k\right) & = & A^{N_{p}}\xi \left(k\right)+A^{N_{p}-1}B\Delta u\left(k|k\right)+A^{N_{p}-2}B\Delta u\left(k+1|k\right)\\ & + & \ldots +A^{N_{p}-N_{c}}B\Delta u\left(k+N_{c}-1|k\right) \end{array}\right.,$$

where $N_{p}$ is the predict horizon, $N_{c}$ is the control horizon, and $N_{c}\leq N_{p}$ that means Δu(k+j|k)=0 when $j\geq N_{c}$. Then, the predicted outputs can be expressed as follows:

(30) $$\left\{ \begin{array}{lll} y\left(k+1|k\right) & = & CA\xi \left(k\right)+CB\Delta u\left(k|k\right)\\ y\left(k+2|k\right) & = & CA^{2}\xi \left(k\right)+CAB\Delta u\left(k|k\right)+CB\Delta u\left(k+1|k\right)\\ y\left(k+3|k\right) & = & CA^{3}\xi \left(k\right)+CA^{2}B\Delta u\left(k|k\right)+CAB\Delta u\left(k+1|k\right)+CB\Delta u\left(k+2|k\right)\\ & \vdots & \\ y\left(k+N_{p}|k\right) & = & CA^{N_{p}}\xi \left(k\right)+CA^{N_{p}-1}B\Delta u\left(k|k\right)+CA^{N_{p}-2}B\Delta u\left(k+1|k\right)\\ & + & \ldots +CA^{N_{p}-N_{c}}B\Delta u\left(k+N_{c}-1|k\right) \end{array}\right..$$

To reduce the notational complexity, the predicted outputs can be expressed as:

(31) Y(k)=Φξ(k)+ΘΔU(k),

in which:

$$\begin{array}{l} Y\left(k\right)=\left[\begin{array}{c} y\left(k+1|k\right)\\ y\left(k+2|k\right)\\ \vdots \\ y\left(k+N_{p}|k\right) \end{array}\right],\Delta U\left(k\right)=\left[\begin{array}{c} \Delta u\left(k|k\right)\\ \Delta u\left(k+1|k\right)\\ \vdots \\ \Delta u\left(k+N_{c}-1|k\right) \end{array}\right]\\ \Phi =\left[\begin{array}{c} CA\\ CA^{2}\\ CA^{3}\\ \begin{array}{l} \vdots \\ CA^{N_{p}} \end{array} \end{array}\right],\Theta =\left[\begin{array}{ccccc} CB & 0 & 0 & \ldots & 0\\ CAB & CB & 0 & \ldots & 0\\ CA^{2}B & CAB & CB & \ldots & 0\\ \vdots & \vdots & \vdots & \ddots & \vdots \\ CA^{N_{p}-1}B & CA^{N_{p}-2}B & CA^{N_{p}-3}B & \ldots & CA^{N_{p}-N_{c}}B \end{array}\right] \end{array}.$$

The objective of the lateral controller is to track the desired path accurately and smoothly. Therefore, define the cost function as:

(32) $$J\left(\xi \left(k\right),\Delta U_{k}\right)=\sum_{i=1}^{N_{p}}{\left\|y\left(k+i|k\right)-y_{ref}\left(k+i|k\right)\right\|}_{Q}^{2}\;+\sum_{i=0}^{N_{c}-1}{\left\|\Delta u\left(k+i|k\right)\right\|}_{R}^{2},$$

where weight matrix Q is symmetric semi-positive definite, R is symmetric positive definite.

Substituting Equation (31) into Equation (32), the cost function can be rewritten as:

(33) $$\begin{array}{lll} J\left(\xi \left(k\right),\Delta U_{k}\right) & = & {\left(Y\left(k\right)-Y_{ref}\left(k\right)\right)}^{T}Q\left(Y\left(k\right)-Y_{ref}\left(k\right)\right)+\Delta U^{T}\left(k\right)R\Delta U\left(k\right)\\ & = & {\left(E+\Theta \Delta U\left(k\right)\right)}^{T}Q\left(E+\Theta \Delta U\left(k\right)\right)+\Delta U^{T}\left(k\right)R\Delta U\left(k\right)\\ & = & \Delta U^{T}\left(k\right)\left(\Theta^{T}Q\Theta +R\right)\Delta U\left(k\right)+2E^{T}\left(k\right)Q\Theta \Delta U\left(k\right)+E^{T}\left(k\right)QE\left(k\right) \end{array},$$

where E(k)=Φξ(k), and $E^{T}\left(k\right)QE\left(k\right)$ is constant, then the cost function is expressed as:

(34) $$J\left(\xi \left(k\right),\Delta U_{k}\right)=\frac{1}{2}\Delta U^{T}\left(k\right)H\Delta U\left(k\right)+g^{T}\Delta U\left(k\right),$$

where hessian matrix $H=2\left(\Theta^{T}Q\Theta +R\right)$, and gradient matrix $g=2\Theta^{T}QE\left(k\right)$.

Considering the constraint $u_{\min}\leq u\left(k\right)\leq u_{\max}$ on the steering angle, the control input sequence U(k) and control increment sequence ΔU(k) satisfy the equation:

(35) U(k)=MΔU(k)+Γu(k−1),

in which:

$$M=\left[\begin{array}{cccc} I & 0 & \ldots & 0\\ I & I & \ldots & 0\\ \vdots & \vdots & \ddots & 0\\ I & I & I & I \end{array}\right],\Gamma =\left[\begin{array}{c} I\\ I\\ \vdots \\ I \end{array}\right],U\left(k\right)=\left[\begin{array}{c} u\left(k|k\right)\\ u\left(k+1|k\right)\\ \vdots \\ u\left(k+N_{c}-1|k\right) \end{array}\right].$$

Considering the constraint $\Delta u_{\min}\leq \Delta u\left(k\right)\leq \Delta u_{\max}$ on steering angle increment, we can have:

(36) $$\Delta U_{\min}\leq \Delta U\left(k\right)\leq \Delta U_{\max}.$$

Considering the outputs constraint $y_{\min}\leq y\leq y_{\max}$, the predicted output sequence satisfies:

(37) $$Y_{\min}\leq \Phi \xi \left(k\right)+\Theta \Delta U\left(k\right)\leq Y_{\max}.$$

Considering the states soft constraint of slip angle of front axle $\alpha_{f}{}_{\min}-\varsigma \leq \alpha_{f}\leq \alpha_{f}{}_{\max}+\varsigma$, the constraint is stated as:

(38) $$\begin{array}{l} M\Delta U\left(k\right)\leq S\left(k\right)-\alpha_{\min}\left(k\right)+\Gamma u\left(k-1\right)\\ -M\Delta U\left(k\right)\leq -S\left(k\right)+\alpha_{\max}\left(k\right)-\Gamma u\left(k-1\right) \end{array},$$

in which:

$$S\left(k\right)=\left[\begin{array}{c} \beta +\frac{l_{f}\gamma }{v_{x}}\\ \beta +\frac{l_{f}\gamma }{v_{x}}\\ \vdots \\ \beta +\frac{l_{f}\gamma }{v_{x}} \end{array}\right]\in {\mathbb{R}}^{{}_{N_{c}\times 1}},\alpha_{\min}\left(k\right)=\left[\begin{array}{c} \alpha_{f}{}_{\min}-\varsigma \\ \alpha_{f}{}_{\min}-\varsigma \\ \vdots \\ \alpha_{f}{}_{\min}-\varsigma \end{array}\right]\in {\mathbb{R}}^{{}_{N_{c}\times 1}},\alpha_{\max}\left(k\right)=\left[\begin{array}{c} \alpha_{f}{}_{\max}+\varsigma \\ \alpha_{f}{}_{\max}+\varsigma \\ \vdots \\ \alpha_{f}{}_{\max}+\varsigma \end{array}\right]\in {\mathbb{R}}^{{}_{N_{c}\times 1}}.$$

Combining Equations (35)–(38), the constraints of control increment sequence ΔU(k) can be written as:

(39) $$\left[\begin{array}{c} M\\ -M\\ I\\ -I\\ \Theta \\ -\Theta \\ -M\\ M \end{array}\right]\Delta U\left(k\right)\leq \left[\begin{array}{c} U_{\max}-\Gamma u\left(k-1\right)\\ -U_{\min}+\Gamma u\left(k-1\right)\\ \Delta U_{\max}\\ -\Delta U_{\min}\\ Y_{\max}-\Phi \xi \left(k\right)\\ -Y_{\min}+\Phi \xi \left(k\right)\\ S\left(k\right)-\alpha_{\min}\left(k\right)+\Gamma u\left(k-1\right)\\ -S\left(k\right)+\alpha_{\max}\left(k\right)-\Gamma u\left(k-1\right) \end{array}\right],$$

where $U_{\max},U_{\min},\Delta U_{\max},\Delta U_{\min}\in {\mathbb{R}}^{{}_{N_{c}\times 1}}$ that consist of $N_{c}$ piece of $u_{\max}$,$u_{\min}$,$\Delta u_{\max}$,$\Delta u_{\min}$, respectively, similarly, $Y_{\max},Y_{\min}\in {\mathbb{R}}^{{}_{N_{p}\times 2}}$ that consist of $N_{p}$ piece of $y_{\max}$,$y_{\min}$, respectively.

The final optimization problem takes the form:

(40) $$\begin{array}{l} \underset{\Delta U\left(k\right)\;\varsigma }{\min}J\left(\xi \left(k\right),\Delta U_{k},\varsigma \right)=\frac{1}{2}\left[\begin{array}{cc} \Delta U^{T}\left(k\right) & \varsigma \end{array}\right]H\left[\begin{array}{c} \Delta U\left(k\right)\\ \varsigma \end{array}\right]+g^{T}\left[\begin{array}{c} \Delta U\left(k\right)\\ \varsigma \end{array}\right]\\ \;s.t.\;\;\mathrm{Equation}(39) \end{array},$$

where the hessian matrix augmented as $H=\left[\begin{array}{cc} 2\left(\Theta^{T}Q\Theta +R\right) & 0\\ 0 & \rho \end{array}\right]$, the gradient matrix augmented as $g=\left[\begin{array}{cc} 2\Theta^{T}QE\left(k\right) & 0 \end{array}\right]$, ς is the slack variable, and ρ is the weight of slack variable.

#### 5.1.3. Quadratic Program Solver

For the quadratic program representable problem as shown in Equation (40), CVXGEN provides a custom, high-speed solver that is library-free C code, which make an online solution as fast as possible. The optimization resolves each time step, as is standard with MPC, and the first element $\Delta u^{*}(k|k)$ of optimal solution is applied to the system, and the feedback control law at time k is finally obtained:

(41) $$u(k|k)=u(k-1)+\Delta u^{*}(k|k).$$

### 5.2. ADRC Controller

Since active disturbance rejection control has strong robustness to system uncertainties and external disturbances, and can be implemented easily, so the nonlinear ADRC is applied to design the lateral controller in this paper. The ADRC is evolved from proportional-integral-derivative (PID) and it inherits the core idea of PID that makes it such a success: the error driven, rather than model-based [15].

#### 5.2.1. ADRC Theoretical Basis

As shown in Figure 3, the nonlinear ADRC consists of three components: the tracking differentiator (TD), extended state observer (ESO), and nonlinear feedback combination (NFC). The TD provides the fastest tracking of the input signal and its derivative, and also arranges the transition process; the ESO considers the total disturbance as a new state variable and gives its estimation; the nonlinear feedback provides surprisingly better results than linear feedback, which play an important role in the ADRC framework.

Consider a single input and single output nonlinear n-order dynamic system:

(42) $$\left\{ \begin{array}{l} x^{\left(n\right)}=f(x,\dot{x},\ldots ,x^{\left(n-1\right)},w,t)+bu\\ y=x \end{array}\right.,$$

where y is the output, u is the input, w is the external disturbance, and $f(x,\dot{x},\ldots ,x^{\left(n-1\right)},w,t)$ is a multivariable function of both the states and external disturbances, as well as time. The objective here is to make y track reference signal $v_{0}\left(t\right)$ using u as the manipulative variable.

To provide the fastest tracking of $v_{0}\left(t\right)$ and get its derivatives, the TD in discrete-time implementation is written as:

(43) $$\left\{ \begin{array}{l} v_{1}\left(k+1\right)=v_{1}\left(k\right)+hv_{2}\left(k+1\right)\\ v_{2}\left(k+1\right)=v_{2}\left(k\right)+hv_{3}\left(k+1\right)\\ \vdots \\ v_{n}\left(k+1\right)=v_{n}\left(k\right)+hu\\ u=fhan(v_{1},v_{2},\ldots ,v_{n},r_{0},h_{0}) \end{array}\right.,$$

where h is the sampling period, fhan(⋅) is the time-optimal solution function, $r_{0}$ and $h_{0}$ are controller parameters.

$f(x,\dot{x},\ldots ,x^{\left(n-1\right)},w,t)$ is denoted as the total disturbance which is something needed to overcome in the context of feedback control. Introducing an additional state variable $x_{n+1}=f(x,\dot{x},\ldots ,x^{\left(n-1\right)},w,t)$, and let ${\dot{x}}_{n+1}=d(t)$, with d(t) unknown. The dynamic system is now described as:

(44) $$\left\{ \begin{array}{l} {\dot{x}}_{1}=x_{2}\\ {\dot{x}}_{2}=x_{3}\\ \vdots \\ {\dot{x}}_{n}=x_{n+1}+bu\\ {\dot{x}}_{n+1}=d(t)\\ y=x_{1} \end{array}\right.,$$

where is always observable. Then, we can construct an extended state observer in the form of:

(45) $$\left\{ \begin{array}{l} e=z_{1}-y\\ {\dot{z}}_{1}=z_{2}-\beta_{1}g_{1}(e)\\ {\dot{z}}_{2}=z_{3}-\beta_{2}g_{2}(e)\\ \vdots \\ {\dot{z}}_{n}=z_{n+1}-\beta_{n}g_{n}(e)+bu\\ {\dot{z}}_{n+1}=-\beta_{n+1}g_{n+1}(e) \end{array}\right.,$$

where $\beta_{i}(i=1,2,\ldots ,n+1)$ are the observer gains and $g_{i}(e)(i=1,2,\ldots ,n+1)$ are nonlinear function of observation error e.

Finally, the control law using nonlinear feedback combination is proposed as:

(46) $$u_{0}=\frac{k_{1}fal(e_{1},\alpha_{1},\delta )+k_{2}fal(e_{2},\alpha_{2},\delta )+\ldots +k_{n}fal(e_{n},\alpha_{n},\delta )-z_{n+1}}{b},$$

where $k_{i}(i=1,2,\ldots ,n)$ are controller gains, $e_{i}(i=1,2,\ldots ,n)$ are the tracking error of state variable, defined as $e_{i}=v_{i}-z_{i}$,fal(⋅) is a nonlinear function that defined as follows:

(47) $$\mathrm{fal}(e,\alpha ,\delta )=\left\{ \begin{array}{l} \begin{array}{cc} {\left|e\right|}^{\alpha }\mathrm{sign}(e), & \;\left|e\right|>\delta \end{array}\\ \begin{array}{cc} e/\delta^{1-\alpha }, & \left|e\right|\leq \delta \end{array} \end{array}\right..$$

It should be pointed out that the controller coefficients are not dependent on the mathematical model of the plant, thus making ADRC largely model independent.

#### 5.2.2. Application of ADRC

For the ADRC method, it is not suitable to process the single input multiple output coupling problem. Therefore, we only consider the lateral error stabilization in the ADRC scheme design. According to the lateral vehicle dynamics Equation (5), we can have:

(48) $${\ddot{y}}_{e}=f\left(y_{e},{\dot{y}}_{e},w,t\right)+\left(\frac{2C_{\alpha f}}{m}+\frac{2C_{\alpha f}l_{f}l_{p}}{I_{z}}\right)\delta_{f},$$

where:

$$\begin{array}{l} f\left(y_{e},{\dot{y}}_{e},w,t\right)=v_{x}^{2}\kappa -v_{x}\gamma -v_{x}\left[\frac{-2(C_{\alpha f}+C_{\alpha }{}_{r})}{mv_{x}}\beta +\left(\frac{-2(C_{\alpha f}l_{f}-C_{\alpha }{}_{r}l_{r})}{mv_{x}^{2}}-1\right)\gamma \right]\\ \;-l_{p}\left[\frac{-2(C_{\alpha f}l_{f}-C_{\alpha }{}_{r}l_{r})}{I_{z}}\beta +\frac{-2(C_{\alpha f}l_{f}^{2}+C_{\alpha }{}_{r}l_{r}^{2})}{I_{z}v_{x}}\gamma \right] \end{array}.$$

Rewrite the equation as the state space form:

(49) $$\left\{ \begin{array}{l} {\dot{x}}_{1}=x_{2}\\ {\dot{x}}_{2}=x_{3}+bu\\ {\dot{x}}_{3}=d(t)\\ y=x_{1} \end{array}\right.,$$

where $x_{1}=y_{e},x_{2}={\dot{y}}_{e},x_{3}=f\left(x_{1},x_{2},w,t\right),b=\frac{2C_{\alpha f}}{m}+\frac{2C_{\alpha f}l_{f}l_{p}}{I_{z}},u=\delta_{f}$.

The ESO for lateral controller is designed as:

(50) $$\left\{ \begin{array}{l} e=z_{1}-y\\ {\dot{z}}_{1}=z_{2}-\beta_{1}g_{1}(e)\\ {\dot{z}}_{2}=z_{3}-\beta_{2}g_{2}(e)+bu\\ {\dot{z}}_{3}=-\beta_{3}g_{3}(e) \end{array}\right..$$

The control law using nonlinear feedback combination can be expressed as:

(51) $$\delta_{f}=\frac{k_{1}fal(e_{1},\alpha_{1},\delta )+k_{2}fal(e_{2},\alpha_{2},\delta )+k_{3}fal(e_{3},\alpha_{3},\delta )-z_{3}}{b}.$$

### 5.3. Pure Pursuit Controller

**Figure 4:**
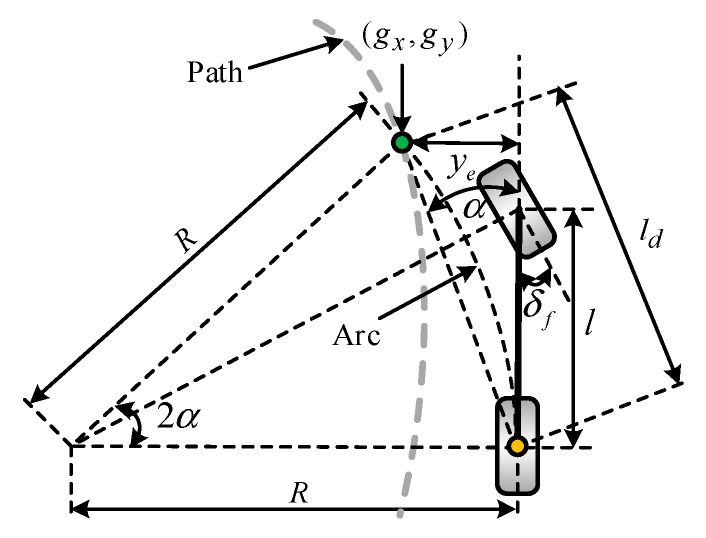
Diagram of the pure pursuit method.

The pure pursuit method (PPM) is used as benchmark, shown in Figure 4. Its basic principle is to make the central control point of the rear axle reach the target point $\left(g_{x},g_{y}\right)$ along an arc by controlling the steering radius of an autonomous vehicle.

Based on the simple geometric bicycle model of an Ackerman steered vehicle, the pure pursuit control law is given as:

(52) $$\delta_{f}=\arctan(\frac{2l\sin(\alpha )}{l_{d}}),$$

where α is the angle between the vehicle heading vector and the look-ahead vector, l is the wheel base, and $l_{d}$ is the look-ahead distance.

## 6. Results and Discussion

In order to evaluate and compare the performance of the proposed path following controllers, a series of simulation experiments have been conducted. The main parameters of the autonomous vehicle are given as Table 1 which are taken from an actual prototype vehicle.

### 6.1. Case A: Speed Tracking

**Figure 5:**
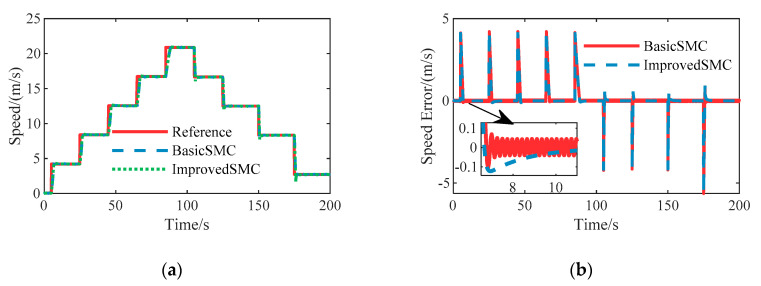
System responses to stair step reference speed on the straight road: (**a**) Speed tracking results; (**b**) Speed tracking error.

**Figure 6:**
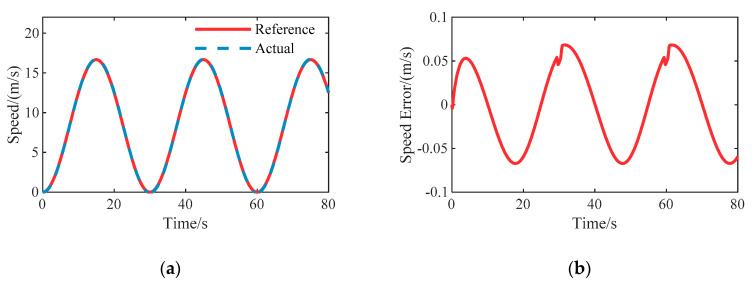
System responses to sinusoidal reference speed on the straight road: (**a**) Speed tracking results; (**b**) The resulting speed tracking error.

To validate the effectiveness of the proposed longitudinal speed tracking controller, stair step and sinusoidal tests have been carried out respectively, and simulation results are shown in Figure 5 and Figure 6. In the stair step case, we compared the improved sliding mode control with the basic sliding mode control to verify the performance improvement. As shown in Figure 5b, the speed error of basic sliding mode control has an obvious chattering effect near zero, which would cause a drastic change in the control input due to the discontinuous sign function. The improved sliding mode control shows a smooth action. It can be seen that the steady state error is almost zero and the overshoot is small. Meanwhile, the mean rise time is about 1.327 s. In the sinusoidal condition, the transient performance of the improved sliding mode speed controller is good. Therefore, the proposed speed tracking controller can provide an excellent performance for longitudinal speed control during the process of path following.

### 6.2. Case B: Skid Pad Test

**Figure 7:**
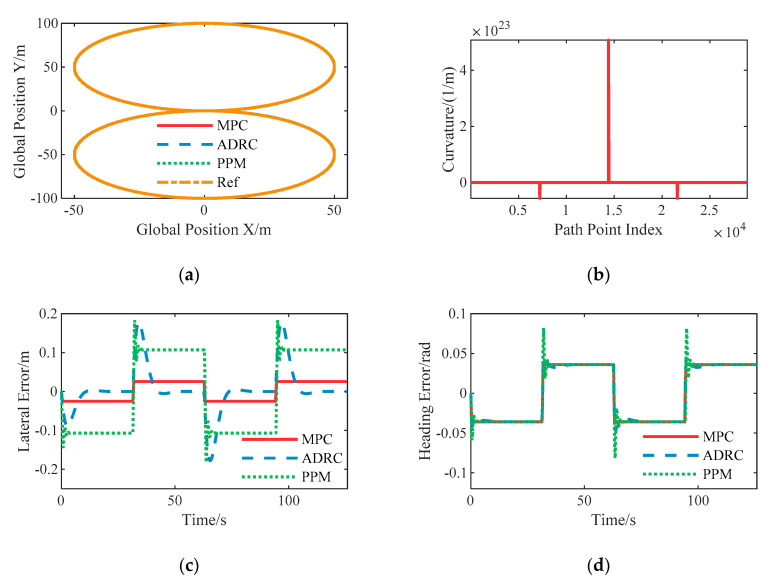
Simulation results of the skid pad test with constant speed 10 m/s: (**a**) The spatial path of the vehicle following the given path; (**b**) Curvature of the given path; (**c**) The resulting lateral error; (**d**) The resulting heading angle error.

To illustrate the steady state characteristics of the proposed lateral controllers for autonomous vehicles, the skid pad test was chosen. As shown as Figure 7a, it consists of two circular paths that intersect at a tangent point. Meanwhile, it includes a point with discontinuous curvature where vehicles transition from one circle to another, which can provide insight into robustness to path curvature.

Figure 7 demonstrates the responses of the designed lateral controllers. It can be seen that the steady state lateral error of the nonlinear ADRC controller is nearly zero and the steady state lateral error of LPV-MPC is about 0.0254 m, while the pure pursuit method-based controller suffers from higher steady state error. Besides, both the nonlinear ADRC and PPM controllers suffer from higher overshoot, in this case due to discontinuous curvature at the tangent point.

### 6.3. Case C: Double Lane Change Test

**Figure 8:**
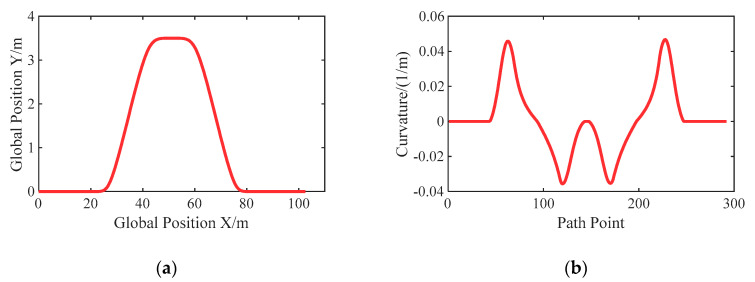
Setting of the double lane change test case: (**a**) Path; (**b**) Curvature.

To investigate the transient response capability of the autonomous vehicle under the conditions of variable and large curvature, the double lane change path is designed, referring to the ISO 3888-2:2002 obstacle avoidance case, as shown in Figure 8.

#### 6.3.1. Constant 5 m/s with the Given Path

In this case, the autonomous vehicle was set to pass through the test area at a constant longitudinal speed of 5 m/s, and the corresponding maximum lateral acceleration is about 1.2 m/s^2^.

**Figure 9:**
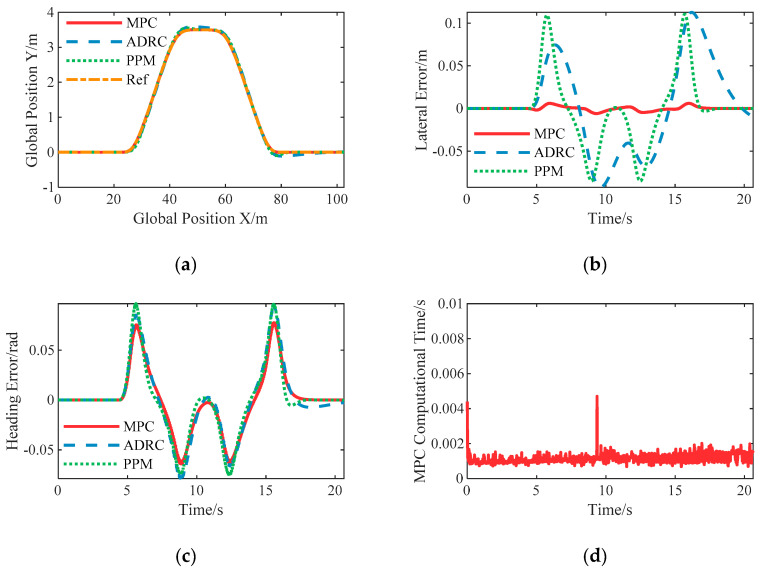
Simulation results of double lane change with constant speed 5 m/s: (**a**) The spatial path of the vehicle following the given path; (**b**) The resulting lateral error; (**c**) The resulting heading angle error; (**d**) The computational time of the proposed LPV-MPC controller.

The control results of the proposed lateral controllers are shown in Figure 9, based on which the control accuracy is then compared. The nonlinear ADRC and PPM controllers achieve almost the similar tracking accuracy in lateral error, while the LPV-MPC had an excellent performance that the maximum lateral error is 0.0061 m. The heading error of the LPV-MPC controller is the smallest, followed by the nonlinear ADRC and PPM controllers. As shown in Figure 9d, using the CVXGEN solver and computer with Intel i7-4770 CPU, the mean computation time of LPV-MPC is about 0.0012 s when predicting 20 steps that the sampling time is 0.02 s, while the others are more computationally efficient than the LPV-MPC controller.

#### 6.3.2. Constant 10 m/s with the Given Path

The desired longitudinal speed is set to 10 m/s in this case, and the corresponding maximum lateral acceleration is about 4 m/s^2^ so that the tires are still in the linear zone.

**Figure 10:**
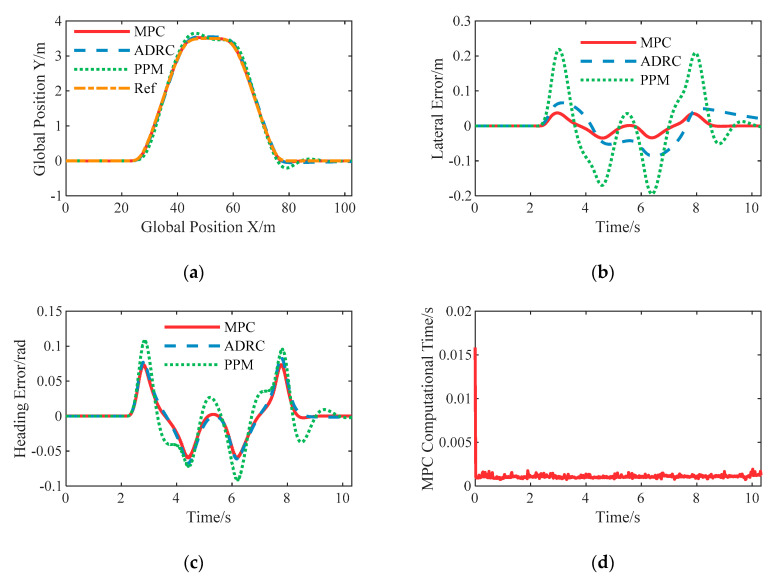
Simulation results of double lane change with constant speed 10 m/s: (**a**) The spatial path of the vehicle following the given path; (**b**) The resulting lateral error; (**c**) The resulting heading angle error; (**d**) The computational time of the proposed LPV-MPC controller.

The responses are presented in Figure 10. Similar to the case of 5 m/s, the LPV-MPC controller has the best transient performance, but the lateral error increases as the vehicle speed increases that the PPM controller has the same feature. It should be noted here that the lateral error of the nonlinear ADRC controller does not change significantly when the vehicle speed increases. This shows the robustness of the nonlinear ADRC controller to the uncertainties of model parameters.

#### 6.3.3. Constant 15 m/s with the Given Path

The desired longitudinal speed is set to 15 m/s in this case, and the corresponding maximum lateral acceleration is about 8 m/s^2^ so that the tires show highly nonlinear characteristic.

**Figure 11:**
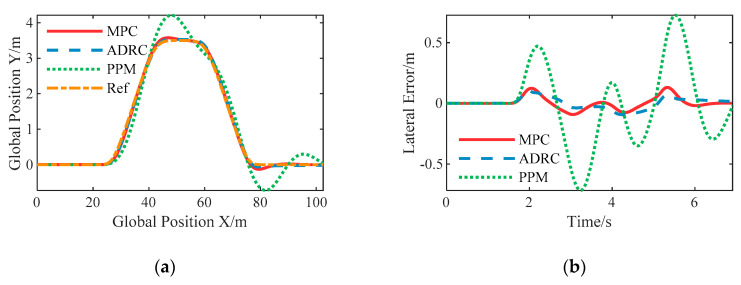

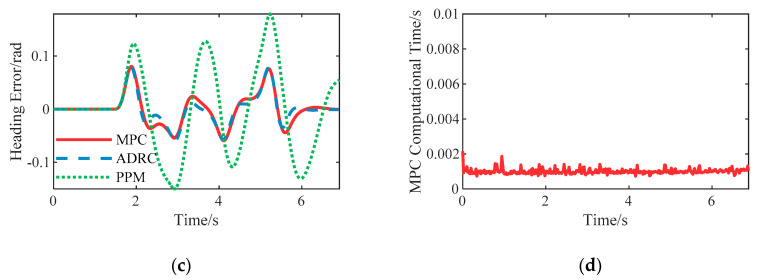
Simulation results of double lane change with constant speed 15 m/s: (**a**) The spatial path of the vehicle following the given path; (**b**) The resulting lateral error; (**c**) The resulting heading angle error; (**d**) The computational time of the proposed LPV-MPC controller.

The simulation results are shown in Figure 11. In this case, the performance of the PPM controller deteriorates significantly, with a maximum lateral error of 0.7258 m and obvious oscillation as well. The nonlinear ADRC controller still keeps stable to the vehicle speed even though large lateral acceleration, which is further verified that the nonlinear ADRC has strong robustness to parametric uncertainties. However, the transient performance of the LPV-MPC controller is worse than the nonlinear ADRC because the proposed LPV-MPC controller adopted a linear model and the actual vehicle dynamics is more nonlinear. The mean calculation time of the LPV-MPC controller is basically the same in these three double lane change tests.

### 6.4. Comprehensive Evaluation

As shown in Table 2, the maximum and root mean square values of lateral displacement error and heading angle error in the double lane change scenario are given, which provides a perspective of comparative analysis for the proposed path following schemes. When the constant test speed is no more than 10 m/s, the MPC has satisfactory control performance that the maximum lateral errors are 0.0061 m and 0.0372 m in test 1 and 4 respectively, far less than the nonlinear ADRC and PPM methods. However, the nonlinear ADRC shows the best performance in path following and the lateral error of MPC increases significantly due to the strong nonlinearity of tires in 15 m/s double lane change tests, while the PPM approach is not applicable because it only considers geometry but ignores vehicle dynamics. From the aspect of tracking performance, the nonlinear ADRC has strong robustness to speed variation. The heading angle error of each method and test is similar except for the PPM method at 15 m/s. The root mean square error indicates the transient performance during the tracking process, and the statistics show that there is the same trend as the maximum value.

Table 3 summarizes the advantages and drawbacks of each control strategy, it can be seen that none of the proposed controller is perfect and all methods can work well in some applications. MPC shows great potential in the vehicle control application with the development of theory and computing power. Nonlinear ADRC can provide strong robustness to uncertainties. The PPM can be used in parking maneuver and low speed vehicles such as sweepers.

**Figure 12:**
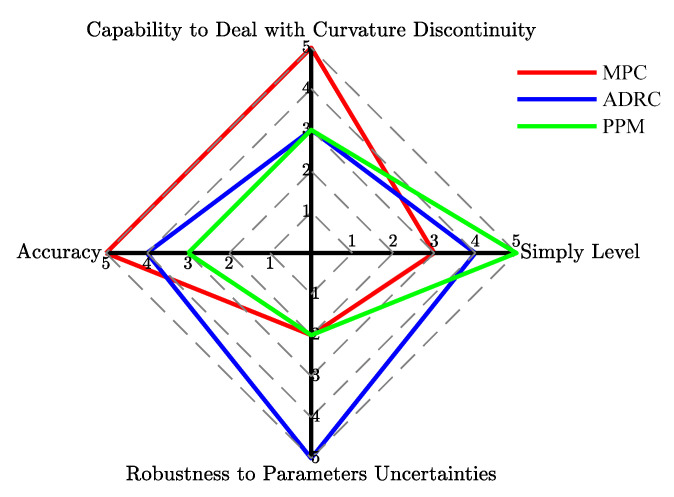
Radar schematic of KPI score of the proposed lateral controllers.

An intuitive evaluation chart is formed by scoring the KPI (Key Performance Indication) of the simulation results, as shown in Figure 12. The score is based on a 5-point scale, with higher scores indicating better performance in that area. Here, the key performance indications include four aspects: path tracking accuracy, robustness to parameters uncertainties, ability to deal with curvature discontinuity, and simplicity of practical application, which represent the four axis terms in Figure 12. It should be noted that scores are evaluated from a perspective of subjective and objective combination. As mentioned above, the LPV-MPC can achieve excellent lateral control precision at low lateral acceleration, and the control accuracy is acceptable even at high lateral acceleration. Due to the predictive ability of MPC, it can also handle the curvature discontinuity of the path. However, MPC is relatively complex to deploy to hardware and requires processing unit of higher computing power. Therefore, the LPV-MPC scores 5, 2, 5, 3 points in these four indicators, respectively. For the nonlinear ADRC, the lateral error and heading error are larger than MPC comprehensively, but it is more robust to the vehicle speed. Meanwhile, the control structure of ADRC is simple and it can be implemented in an embedded vehicle control unit, but the parameters need to be calibrated carefully. Path curvature discontinuity would cause a larger overshoot in the nonlinear ADRC controller. In general, the nonlinear ADRC scores 4, 5, 3, 4 points, respectively. The pure pursuit method only works well in the low speed case but it is the simplest in practical application. Then, the PPM scores 3, 2, 3, 5 points in these indexes, respectively.

For model predictive control, further research is needed to build a good model that tradeoff between high fidelity and simple enough, and robust and stochastic MPC may be the future direction.

## 7. Conclusions

In this paper, three control schemes have been designed, verified, and compared for the path following application of autonomous vehicles. A longitudinal speed tracking controller is proposed based on sliding mode control with nonlinear conditional integrator, and stability analysis is also given. Considering the ability of preview and handling multi-constraints, the first lateral controller is based on the linear parameter varying model predictive control, which shows excellent tracking accuracy in the simulation tests. Though it requires real-time computations to solve the optimization problem, using the CVXGEN can achieve high efficiency to solve the QP problem. Nonlinear active disturbance rejection control is applied in the second lateral controller since ADRC is robust against the parametric uncertainties of vehicles. However, the comprehensive tracking performance is slightly worse than the MPC that was shown in comparison. The lateral controller via the pure pursuit method is used for a benchmark, which performs well in low speed case. The effectiveness and ability of the proposed schemes are validated by simulations. It should be noted that the LPV-MPC controller is relatively sensitive to the parametric uncertainties and the vehicle dynamics is highly nonlinear in large acceleration cases, therefore, the robust nonlinear model predictive control is our future research direction.

## Figures and Tables

**Table 1:** Main parameters of the autonomous vehicle.

Definition	Symbol	Value	Unit
Vehicle mass	m	1381	kg
Distance from COG to the front axle	$l_{f}$	1.117	m
Distance from COG to the rear axle	$l_{r}$	1.188	m
Yaw moment of inertia of the vehicle	$I_{z}$	1833.8	kg/m^2^
Cornering stiffness of the front wheel	$C_{\alpha f}$	30,087	N/rad
Cornering stiffness of the rear wheel	$C_{\alpha r}$	31,888	N/rad
Spin inertia of the wheel	$I_{\omega }$	0.4	kg
Rolling radius of the wheel	R	0.291	m

**Table 2:** Quantitative statistics of lateral error and heading error in the double lane change scenario.

Test No.	Desired Speed	Controller Design	Maximum Lateral Error	RMSE ^1^ of Lateral Error	Maximum Heading Error	RMSE of Heading Error
1	5 m/s	MPC	0.0061	0.0024	0.0776	0.0302
2	5 m/s	ADRC	0.1127	0.0520	0.0941	0.0355
3	5 m/s	PPM	0.1107	0.0403	0.0966	0.0345
4	10 m/s	MPC	0.0372	0.0164	0.0735	0.0275
5	10 m/s	ADRC	0.0872	0.0430	0.0833	0.0305
6	10 m/s	PPM	0.2186	0.0921	0.1080	0.0398
7	15 m/s	MPC	0.1312	0.0504	0.0806	0.0293
8	15 m/s	ADRC	0.1033	0.0456	0.0796	0.0272
9	15 m/s	PPM	0.7258	0.3218	0.1793	0.0819

^1^ Root mean square error.

**Table 3:** Comparison of the lateral control strategies.

Controller Design	Advantages	Drawbacks
LPV-MPC	Can exploit available preview Easy to handles constraints Excellent control accuracy	Requires real-time computations Need a more accurate model Rely on sideslip angle information Sensitive to parametric uncertainties
ADRC	Robust against parameters uncertainties Easy to implement	Controller gains are need to be tuned well to get higher precision of tracking
PPM	Simply enough Performs well when driving slowly or parking	Neglect the vehicle dynamics, so it does not suitable to large lateral acceleration case Suffer from overshoot and steady state error as speed increases
